# Optimization of Sulfurization Process of Cobalt Sulfide and Nitrogen Doped Carbon Material for Boosting the Oxygen Reduction Reaction Catalytic Activity in Alkaline Medium

**DOI:** 10.3389/fchem.2020.00314

**Published:** 2020-04-28

**Authors:** Bing-Ye Song, Sen Yao

**Affiliations:** ^1^Key Laboratory of Thermo-Fluid Science and Engineering of MOE, School of Energy & Power Engineering, Xi'an Jiaotong University, Xi'an, China; ^2^Key Laboratory of New Materials and Facilities for Rural Renewable Energy of MOA, Henan Agricultural University, Zhengzhou, China

**Keywords:** oxygen reduction reaction, metal organic frameworks, non-noble-metal materials, sulfurization, co-based catalyst

## Abstract

In order to reduce fuel cell material cost and promote its application, it is urgent to develop non-noble metal materials to replace platinum as the cathode catalysts in fuel cells. The cobalt sulfide and nitrogen co-doped carbon (S-Co-N/C) materials with metal-organic frameworks as precursors have shown good oxygen reduction reaction (ORR) catalytic activity. Benefiting from the protection of catalytic active sites by sulfur atoms, the stability and alcohol-tolerance of the S-Co-N/C catalyst can be significantly improved. In order to fully understand the effect of the sulfurization process on the properties of the material, zeolite imidazole frameworks (ZIF)-8, and ZIF-67 are used as precursors to prepare a novel material of S-Co-N/C by using a sulfurization-pyrolysis method. Another S-Co-N/C material by using a pyrolysis- sulfurization method is prepared for comparison. The effects of the sulfurization process in the preparation on the morphology, chemical structure, specific surface area, and ORR catalytic activity of the final material are investigated. The experimental results show that the surface of the S-Co-N/C material tends to be rough due to the sulfurization reaction of the metal elements. The porosity of the material is reduced to some extent due to the remaining Zn elements in the final product. Interestingly, some carbon nanotubes are found to be generated on the surface of the S-Co-N/C material because of the synergistic effect of Zn and Co on the carbon material during the pyrolysis process, which is beneficial to accelerate the adsorption of oxygen on the S-Co-N/C surface and the electron transportation during the oxygen reduction reaction. In addition, the generated CoS during the sulfurization process can further protect the Co elements from agglomeration, which can effectively increase the ORR catalytic active sites in the final material. The S-Co-N/C material prepared by the sulfurization-pyrolysis method performs a superior ORR catalytic activity to the one synthesized by the pyrolysis-sulfurization method.

## Introduction

In the past 5 years, the technology of fuel cells has tended to be mature, which has accelerated its industrial development in the transport sector. However, the high cost of fuel cell stacks is still one of the main factors limiting the commercialization of fuel cell applications (Alaswad et al., 2016; Sun et al., 2019). The fuel cell material costs include the cost of bipolar plates, proton exchange membranes, catalysts, gas diffusion layers, and membrane electrode frameworks (Tsuchiya and Kobayashi, 2004). Among them, scarce platinum-based materials are still used as catalysts for the cathode and anode electrochemical reactions in the current application of fuel cells (Whiston et al., 2019). According to the research of Thompson et al. (2018), the catalyst cost accounts for 41% of the stack cost, which is the major reason for the high price of fuel cells. More seriously, due to the increasing scarcity of platinum and the maturity of fuel cell process technology, the cost of platinum-based materials relative to fuel cell system cost would gradual increase (Sun et al., 2011). In order to reduce the cost and achieve sustainable development of fuel cells, it is urgent to develop non-precious metal materials for replacing platinum completely as the catalysts in the electrodes of fuel cells (Meng et al., 2019; Xu et al., 2019; Yang et al., 2019). Especially in the cathodes, the sluggish oxygen reduction reaction (ORR) kinetics make cathode electrodes rely on platinum-based materials with excellent catalytic activity.

At present, carbon materials (such as active carbon, carbon nanotubes, graphene, etc.) with transition metals (such as Co, Fe, Ni, etc.) doped on them have been proven to have good catalytic activity for the oxygen reduction reaction, and are promising candidates for alternating the platinum-based catalysts completely (Zhang Z. et al., 2018; Cheng et al., 2019; Li Z. et al., 2019; Khan et al., 2020). The abundant and cheap transition metals can effectively reduce the cost of the fuel cell materials. Among the developed transition metal-based catalysts, the cobalt sulfides have shown excellent ORR activity, stability, and alcohol-tolerance (Guo et al., 2018; Kumar et al., 2018). In addition, nitrogen doped carbon materials without metal loading have also been found to have ORR catalytic activity (Deng et al., 2017; Kong et al., 2017; Guo et al., 2020). According to the bonding type between N and C, the types of nitrogen can be divided into graphitic N, pyridinic N, pyrrolic N, and oxidized N (Liu et al., 2016). Among them, pyridinic N has been confirmed to be the ORR active site by Guo et al. (2016). Thus, it can be deduced from the above findings that embedding cobalt sulfides into the N-doped carbon materials (S-Co-N/C) can effectively increase the active sites of oxygen reduction reaction. Han et al. (2018) fabricated a core-shell structured ORR catalyst with CoS nanowires as core and N, S doped graphitic carbon as shell. The CoS NWs@NSC material shows an excellent electrocatalytic activity due to the uniform distributed ORR active sites and the interaction between CoS and graphitic carbon.

It must be mentioned that the structure of the component directly determines the mass transfer process in the system, which in turn affects the performance (Jiang et al., 2019; Wang et al., 2020). Therefore, the development of porous materials with controlled structures is an important way to improve the ORR catalytic activity. Metal organic frameworks (MOFs) with three-dimensional porous structures are composed of metal connection points and organic ligands. Due to the advantages of a high specific surface area, regular morphology, and porous structure, MOFs are promising precursors to fabricate metal and nitrogen co-doped materials with high efficient ORR activity (Li L. et al., 2019; Zhang et al., 2019). Zhou et al. (2019) prepared a novel ORR catalyst of NiCo/CN by a one-step pyrolysis of bimetallic MOFs. In the obtained 3D hierarchical NiCo/CN, the bimetallic nanoparticles can be uniformly dispersed in the graphitized N-doped carbon skeleton, which can enhance the ORR activity. As a kind of MOFs, zeolite imidazole framework (ZIF)-67 with cobalt ions as the metal connection points has been widely used to prepare Co-N/C catalysts. Qiao et al. (2020) synthesized a material of Co-NOPC with a 3D ordered porous structure with polystyrene molded ZIF-67 as precursor. The resultant Co-NOPC performs a superior ORR activity to the commercial Pt/C relying on its hierarchical porosity. Lv et al. (2018) used reduced graphene oxide integrated ZIF-67 as precursors to fabricate an excellent ORR catalyst, which shows a close approaching half-wave potential to the Pt/C. A novel graphene warped Co/NC catalyst was obtained by Gao et al. (2020) by the calcination of the ZIF-67 on graphene. The product of Co/NC-Gr exhibited good ORR catalytic activity, durability, and methanol tolerant ability due to the large surface area, high electron conductivity, and high contents of nitrogen.

However, with ZIF-67 as the precursor, the Co elements are easy to agglomerate during the pyrolysis process, which would reduce the specific surface area and oxygen reduction catalytic activity of the obtained materials (Zhang M. et al., 2018). To solve this problem, Hu et al. (2016) used CoS covered ZIF-67 as the precursor to prepare a material of Co-C@Co_9_S_8_ with core-shell structure. The CoS shell can protect the cobalt atoms from agglomeration during the annealing process. The obtained final product performed an outstanding ORR catalytic activity. In the previous study, a material of Co@Co_9_S_8_-N/C was prepared by co-pyrolysis-sulfurization of ZIF-8 and ZIF-67. In this way, the doping content of N and the specific surface area were increased, which is helpful in obtaining a material with an enhanced ORR electrocatalytic activity (Song et al., 2020). According to the above literature research, it can also be shown that the doped sulfur elements in the catalysts can protect the ORR active sites from degeneration, which plays a vital role in improving the stability and durability of the final materials. The sulfurization process during the material preparation process is very important for doping sulfur elements in the product. However, in many S-Co-N/C materials prepared with ZIF-67 as the precursor, the effect of the sulfurization process on the material is still insufficiently understood. Based on this, in this work, by using ZIF-8 and ZIF-67 as precursors, the effects of the sulfurization process on the morphology, chemical composition, specific surface area, and ORR catalytic activity of the final cobalt sulfide and nitrogen doped carbon material are investigated.

## Experimental Method

In this work, with ZIF-8 and ZIF-67 as precursors, two kinds of S-Co-N/C materials were synthesized by changing the sequence of sulfurization and pyrolysis during the preparing process. The micro-morphology, chemical composition, specific surface area, porosity, and ORR catalytic activity of the obtained products were characterized. The uncertainty of the experimental results was analyzed.

### Reagents

The novel ORR catalyst of S-Co-N/C was prepared with the regents of Zn(NO_3_)_2_·6H_2_O (≥99 wt.%, Damao Chemical Reagent Factory), Co(NO_3_)_2_·6H_2_O (≥99 wt.%, Guangdong Guanghua Chemical Factory Co. Ltd.), 2-methylimidazole (Aladdin Industrial Corporation), methanol (≥99.5 wt.%, Tianjin Fuyu Fine Chemical Co. Ltd.), and thioacetamide (≥99 wt.%, Tianjin Kemiou Chemical Reagent Co. Ltd.). Potassium hydroxide (≥85 wt.%, Sichuan Xilong Chemical Co. Ltd.) was used as electrolyte during the ORR activity measurement. The reagents mentioned above were all analytical grade without any further purification. Ultrapure water (18.25 MΩ·cm^−1^ Millipore-Milli-Q) was used to prepare all the aqueous solutions in this work.

### Synthesis of the S-Co-N/C Materials

ZIF-67 and ZIF-8 mixed precursors (ZIFs) were first prepared by co-precipitation at room temperature. Then, a cobalt sulfide and nitrogen co-doped carbon material was prepared by the sulfurization-pyrolysis of ZIFs, marked as S-Co-N/C-I. For comparison, the obtained ZIFs were pyrolyzed first and then sulfurized to obtain another material, which was marked as S-Co-N/C-II. The specific preparation procedures of the two materials are as follows.

1. 14.4 mmol of Zn(NO_3_)_2_·6H_2_O and 9.6 mmol of Co(NO_3_)_2_·6H_2_O were mixed and dissolved in 240 mL of methanol to obtain a solution A. 96 mmol of 2-methylimidazole was dissolved in 80 mL of methanol to obtain a solution B.
2. The coprecipitation reaction was activated by adding the solution A slowly into the solution B at room temperature. During this process, the Co^2+^ and Zn^2+^ in solution A were coordinated with the N atoms in solution B to form ZIFs.
3. The obtained ZIFs mixture was washed with deionized water several times to remove impurities, and then dried at 60°C for 12 h.
4. In order to prepare the S-Co-N/C-I, the as-formed ZIFs were added into the ethanol solution of thioacetamide (TAA) with vigorous stirring, followed by hydrothermally reacting at 120°C. During this process, the CoS and ZnS were formed on the surface of ZIFs. After washing and drying, the particles were finally pyrolyzed at 950°C for 3 h at nitrogen atmosphere to obtain the S-Co-N/C-I.
5. The S-Co-N/C-II was fabricated according to the previous report (Song et al., 2020). In brief, the prepared ZIFs were first annealed from room temperature to 950°C at a heating rate of 3°C·min^−1^ under the protection of nitrogen, and maintained for 3 h to obtain Co-N/C. Afterwards, the Co-N/C particles were sulfurized by hydrothermally reacting with TAA to get the S-Co-N/C-II.

### Characterization Methods for the S-Co-N/C Materials

The microstructure of the obtained S-Co-N/C catalysts directly affect the distribution of the ORR catalytic active sites and the mass transportation in the material. In order to visually describe the micro-morphology of the electro-catalysts, the prepared S-Co-N/C materials were characterized by a scan electron microscopy (SEM, Gemini 500, Zeiss, Germany) and a transmission electron microscopy (TEM, JEM-F200, JEOL, Japan). The accuracy Zn and Co contents in the synthetic S-Co-N/C materials were tested using an inductively coupled plasma-mass spectrometry (ICP-MS, NexION 350D, Perkin Elmer, USA). An X-ray photoelectron spectroscopy (XPS, ESCALAB Xi+, Thermo Fisher, USA) was applied to study the surface elemental composition of the obtained samples. The specific surface area and pore size distribution of the S-Co-N/C materials were explored by an automatic surface and porosity analyzer (BELSORP-Max, MicrotracBEL, Japan).

The ORR catalytic activity of the prepared S-Co-N/C materials was tested by a linear scanning voltammetry (LSV) method with rotating disk electrode (RDE, PINE, USA) in a three-electrode cell assembly. The RDE was connected with an electrochemical workstation (AUTOLAB PGSTAT302N), which was used to control the potential of the working electrode and record the corresponding current. In the three-electrode cell assembly, a glass carbon electrode with a catalyst loading of 196 μg·cm^−2^, a platinum foil, and a Hg/HgO (MMO, 1.0 mol·L^−1^) electrode were applied as the working electrode, counter electrode, and reference electrode, respectively. Equation 1 was used to transform the measured potentials (*vs*. MMO) to the potential vs. reversible hydrogen electrode (RHE).

(1) $$\begin{array}{l} E\left(vs.\text{RHE}\right)=E\left(vs.\text{MMO}\right)+0.0591\times pH+0.098 \end{array}$$

Before the LSV test, in order to get a saturated oxygen environment in the alkaline electrolyte, oxygen was pumped into the 0.1 mol·L^−1^ KOH solution with a rate of 50 standard-state cubic centimeter per minute (sccm) for 30 min. Afterwards, the cyclic voltammetry scan was performed on the catalyst coated working electrode with a rotating speed of 1,600 rpm at a scan speed of 200 mV·s^−1^ between the potential of −0.866 V and 0.334 V (vs. MMO) 50 times to activate the catalyst. Under the same rotating speed of RDE, the LSV curves of the Co-N/C catalysts were then obtained by scanning the potential from 0.334 V to −0.866 V (vs. MMO) at a rate of 10 mV·s^−1^.

### Uncertainty Analysis

Despite repeating every LSV test at least three times to reduce the experimental errors, the possible error sources during the ORR catalytic activity tests still exist due to the measurement errors of the alkaline solution concentration (*C*), the mass of the catalyst loaded on the working electrode (*m*), the area of the glass carbon electrode (*A*), the rotation speed of RDE (*r*), and the potential controlled by the electrochemical workstation (*V*), which were 1, 1.25, 1, 1, and 0.2%, respectively. Based on the uncertainty propagation analysis (Song et al., 2019a,b), as shown in Equation 2, the uncertainty of ORR activity test was calculated to be 2.1%.

(2) $$\begin{array}{l} \frac{\delta I_{\;}}{I}=\sqrt{{\left(\frac{\delta C}{C}\right)}^{2}+{\left(\frac{\delta m}{m}\right)}^{2}+{\left(\frac{\delta A}{A}\right)}^{2}+{\left(\frac{\delta r}{r}\right)}^{2}+{\left(\frac{\delta V}{V}\right)}^{2}} \end{array}$$

## Results and Discussion

The effects of the sulfurization sequence during the preparation of the S-Co-N/C material on the morphology, chemical composition, specific surface area, and porosity of the final material were first explored. Afterwards, the ORR catalytic activity of the obtained S-Co-N/C material was evaluated.

### Effect of Sulfurization on the Microstructure of the S-Co-N/C

**Figure 1:**
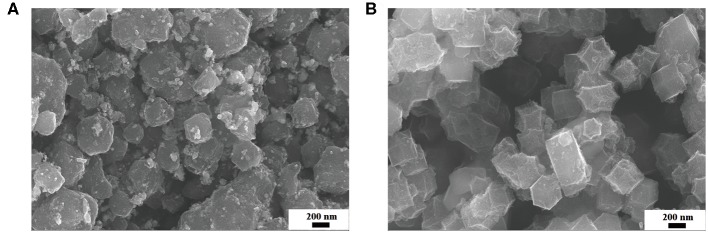
SEM images of **(A)** the S-Co-N/C-I, and **(B)** S-Co-N/C-II.

The SEM and TEM were applied to study the micro-morphology of the S-Co-N/C catalysts. It can be seen from Figure 1 that both of the S-Co-N/C samples derived from MOFs have shown the typical polyhedral morphology. The size of a single S-Co-N/C particle is about 300 nm. However, the surface of the S-Co-N/C-II (Figure 1B) is smoother than that of the S-Co-N/C-I (Figure 1A), which can be attributed to the following aspects. On one hand, the Zn elements in the ZIFs were vapored out during the prior pyrolysis process at 950°C, which reduced the metal sulfurization degree of the final material of the S-Co-N/C-II. In addition, the intermediate product of Co-N/C formed after the pyrolysis was very stable, which slowed the metal sulfurization reaction kinetics. On the other hand, during the synthetic process of S-Co-N/C-I, the prior sulfurization can promote the reaction of sulfur elements with Co and Zn to form CoS and ZnS, which can increase the roughness of the material after pyrolysis. Interestingly, as shown in Figure 1A, some protrusions with tubular structure are found to have formed on the surface of the S-Co-N/C-I.

**Figure 2:**
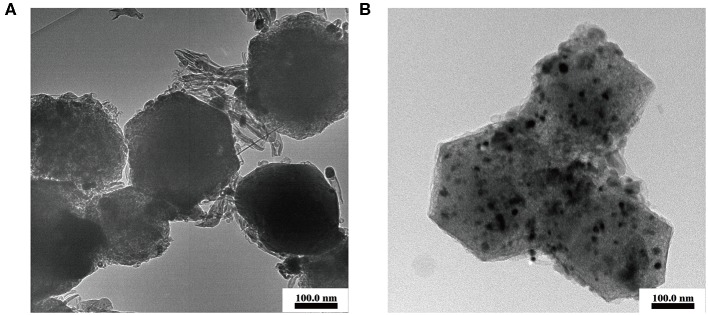
TEM images of **(A)** the S-Co-N/C-I and **(B)** S-Co-N/C-II.

To further study the effect of the sulfurization process on the microstructure of the S-Co-N/C catalyst, TEM images of the obtained two samples in Figure 2 were also tested. The cobalt elements of the S-Co-N/C-I in Figure 2A can be homogeneously embedded into the carbon skeleton, while the cobalt elements of the S-Co-N/C-II, as shown in Figure 2B, are easily aggregated to black particles. During the pyrolysis of the S-Co-N/C-I preparation process, the CoS and ZnS shells formed by the sulfurization of the ZIFs can protect the internal Co elements from agglomeration and promote the uniform distribution of Co-C in the final material. The tubular protrusions on the surface of the S-Co-N/C-I are proved to be nitrogen-doped highly graphited carbon nanotubes by the TEM image in Figure 2A. The generated carbon nanotubes can be attributed to the synergistic effect of Zn and Co on the carbon material during the carbonization of the ZIFs. During the preparation process of the S-Co-N/C-I, a number of the Zn elements were retained inside the material due to the prior sulfurization. In the following pyrolysis process, the carbon on the surface of the material was induced by the Zn and Co elements to generate the carbon nanotubes. Benefiting from the formed carbon nanotubes, the electron transportation in the S-Co-N/C-I during the electrocatalytic oxygen reduction reaction can be accelerated.

In order to get the accurate content of the metal elements in the S-Co-N/C catalysts, 15 mg of the obtained catalyst was first digested with a mixture of concentrated hydrochloric acid and concentrated nitric acid (volume ratio of 1: 3) at 130°C for 1 h to dissolve the metals of the material in a liquid. After filtration and volume adjustment to 50 mL, the metal elements in the liquid can be quantitatively tested by a ICP-MS. The contents of Zn and Co in the final materials of S-Co-N/C are shown in Table S1. The Co contents of the obtained two ORR catalysts are almost the same, maintaining between 12 and 13 wt.%. In the S-Co-N/C-I prepared by the sulfurization-pyrolysis process, 2.87 wt.% of Zn remains. This is because the ZnS with a boiling point of 1,185°C formed on the surface of the S-Co-N/C-I during the sulfurization process cannot be vapored out at the pyrolysis temperature of 950°C. However, the Zn atoms in the S-Co-N/C-II can be almost evaporated during the prior pyrolysis process, leading to only 0.2 wt.% of Zn remaining in the final product.

**Figure 3:**
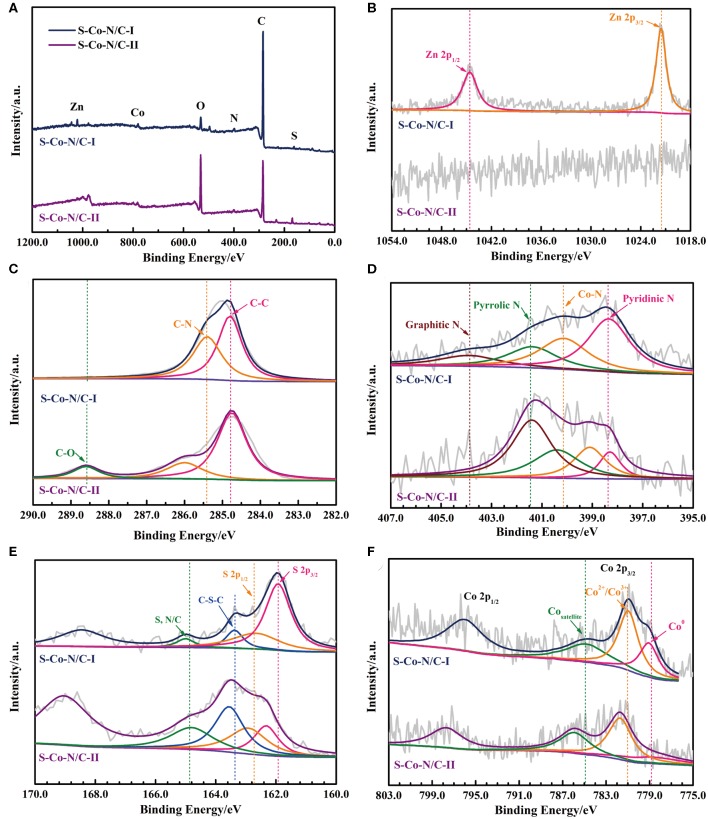
**(A)** XPS survey scan, High resolution-XPS spectrum of **(B)** Zn 2p, **(C)** C 1s, **(D)** N 1s, **(E)** S 2p, and **(F)** Co 2p in the S-Co-N/C materials.

The XPS results of the two S-Co-N/C catalysts are shown in Figure 3 to further investigate the chemical states of the elements on the surface of materials. The characteristic peaks of O, Zn, C, N, S, and Co elements are detected in the XPS survey scan in Figure 3A. The apparent characteristic peak of O in Figure 3A can be attributed to the introduced oxygen from the air into the materials during the sulfurization process. However, the characteristic peak of O in the S-Co-N/C-I XPS spectrum is not as obvious as that in the S-Co-N/C-II spectrum, which can be explained as follows. During the pyrolysis process of the S-Co-N/C-I after sulfurization, the induced oxygen was first reacted with the metal atoms to form metal oxides. As the pyrolysis temperature increased, the metal oxides would be gradually reduced to metal atoms by the generated carbon. At the same time, the oxygen-containing species were removed out from the material.

The binding energies at the deconvoluted peaks in the high resolution-XPS (HR-XPS) spectrums of Zn, C, S, N, and Co are listed in Table S2. The two deconvoluted peaks in the high-resolution Zn 2p spectrum of S-Co-N/C-I (Figure 3B) at 1044.6 eV and 1021.5 eV are corresponded to the Zn 2p_1/2_ and Zn 2p_3/2_, respectively, which indicates the residual Zn elements on the surface of the material due to the prior sulfurization. However, because almost all the Zn elements are removed out of the S-Co-N/C-II as demonstrated by the ICP-MS, no obvious peak can be detected in its Zn 2p spectrum. The C 1s spectrum of the S-Co-N/C-I in Figure 3C can be fitted to two peaks of C-C and C-N at the binding energy of 284.8 and 285.4 eV. The 0.6 eV positive shifted C-N peak of the S-Co-N/C-II can be attributed to the transfer of electrons from C to N, indicating that more N atoms have been doped in the carbon skeleton of the S-Co-N/C-II. Since the pyrolysis of ZIF-8 in the precursor is the main nitrogen source of the final S-Co-N/C material, some ZIF-8 precursors were sulfurized during the synthetic process of S-Co-N/C-I, resulting in the reduced content of N in the final product. This phenomenon can also be confirmed from the negative shifted graphitic N and pyrrolic N peaks in the N 1s spectrum of the S-Co-N/C-II (Figure 3D). However, in the N 1s HR-XPS spectrum of S-Co-N/C-I, the pyridinic N peak at the binding energy of 398.3 eV is more obvious than the deconvoluted peaks of other forms of N, which is conducive to enhancing the ORR catalytic activity of the material.

The S 2p spectrum in Figure 3E shows that the fitted two peaks of S 2p_3/2_ and S 2p_1/2_ of the S-Co-N/C-I detected at the binding energy of 161.9 and 162.7 eV are negatively shifted by comparing them with the two peaks of the S-Co-N/C-II, which indicates a stronger interaction between S and metal element in the S-Co-N/C-I. This is because the ZIFs are not as stable as the Co-N/C produced after the pyrolysis of ZIFs. The sulfurization of ZIFs can promote the reaction between sulfur and metal elements. However, due to the presence of Zn atoms in the precursor of ZIFs, the sulfurization process would lead some S elements to react with Zn, resulting in a weakened interaction between S and Co in the final material of S-Co-N/C-I. Therefore, as shown in Figure 3F, the binding energy of Co^2+^/Co^3+^ peak in the Co 2p spectrum of S-Co-N/C-I is more negative than that of S-Co-N/C-II. During the pyrolysis process of the sulfurized ZIFs, the porosity of the material was increased by the evaporation of Zn at 950°C, leading more Co atoms inside the crystals to be exposed on the surface of the S-Co-N/C-I. Thus, the Co^0^ peak at the binding energy of 778.7 eV in the Co 2p spectrum of the S-Co-N/C-I is more obvious than that of the S-Co-N/C-II, which is beneficial for improving the electro-catalytic activity of the material.

**Figure 4:**
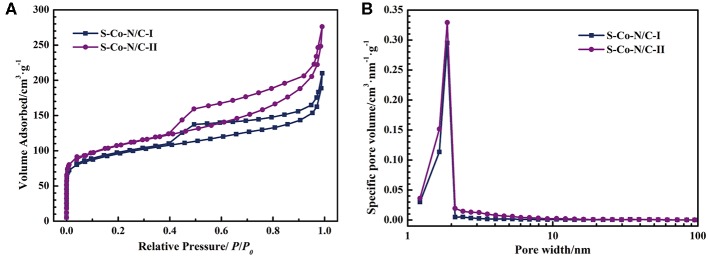
**(A)** Nitrogen adsorption-desorption curves of the S-Co-N/C, **(B)** Pore size distribution curves of the S-Co-N/C.

The nitrogen adsorption-desorption tests were performed on the two materials of S-Co-N/C to explore the effect of the sulfurization process on the specific surface area and porosity of the final product. The nitrogen adsorption curve in Figure 4A shows that the amount of N_2_ adsorbed by the two samples increases rapidly as the relative pressure rises in a low pressure area, indicating that a large number of micropores exist in both of the S-Co-N/C samples. In addition, the hysteresis loop of the nitrogen desorption curve in Figure 4A indicates the presence of mesopores in the two materials.

In the low relative pressure area in Figure 4A, the amount of N_2_ adsorbed by the S-Co-N/C-II is more than that by the S-Co-N/C-I, demonstrating a larger number of micropores in the S-Co-N/C-II. The above finding can also be confirmed by the pore size distribution curve in Figure 4B. Micropores are mainly generated by the evaporation of Zn in ZIFs during pyrolysis. During the preparation of the S-Co-N/C-I, ZnS produced by sulfurization cannot be evaporated by the after pyrolysis process, resulting in a reduced number of micropores in the final material. However, as shown in Table S3, the specific surface area of the S-Co-N/C-I calculated by the Brunauer-Emmet-Teller method is not decreased significantly. This is because the carbon nanotubes generated on the surface of the S-Co-N/C-I are beneficial for the specific surface area increasement of the material.

### ORR Catalytic Activity of the S-Co-N/C

**Figure 5:**
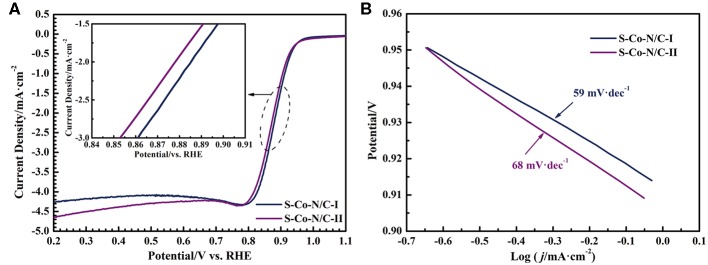
**(A)** LSV curves of the S-Co-N/C and **(B)** the corresponding Tafel plots of the S-Co-N/C.

The LSV measurement in a rotating disk electrode was used to study the influence of sulfurization on the ORR catalytic performance of the S-Co-N/C. By analyzing the LSV curves in Figure 5A, the onset potentials and half-wave potentials of the obtained two materials were obtained, as shown in Table S4. The onset potentials of the S-Co-N/C-I and S-Co-N/C-II are equivalent to 1.009 V (vs. RHE). A higher half-wave potential of the S-Co-N/C-I (0.879 V vs. RHE) reveals that the ORR catalytic activity of the S-Co-N/C-I is superior to that of the S-Co-N/C-II (0.868 V vs. RHE), which can also be demonstrated by the Tafel slopes of the two materials in Figure 5B. The Tafel slope of the S-Co-N/C-I (59 mV·dec^−1^) is lower than that of the S-Co-N/C-II (68 mV·dec^−1^), which proves a lower overpotential of the oxygen reduction reaction catalyzed by the S-Co-N/C-I.

The enhanced ORR electro-catalytic activity of S-Co-N/C-I can be attributed to the following three aspects. (1) The CoS and ZnS shells formed by the sulfurization of ZIFs can protect the Co atoms inside from agglomeration during the pyrolysis process, which make a uniform distribution of Co in the S-Co-N/C-I. The uniformly dispersed Co-C can promote the transportation of electrons from carbon to oxygen, leading to a reduced adsorption free energy of oxygen on the surface of the S-Co-N/C-I. (2) As confirmed by the XPS test, the content of pyridinic N in the S-Co-N/C-I is higher than that of other forms of N, which means an increase of ORR active sites in the S-Co-N/C-I. (3) The carbon nanotubes formed on the surface of the S-Co-N/C-I due to the synergistic effect of Co and Zn on the carbon material can not only enhance the adsorption of oxygen on the catalyst, but also accelerate the electron transfer during the oxygen reduction reaction.

**Figure 6:**
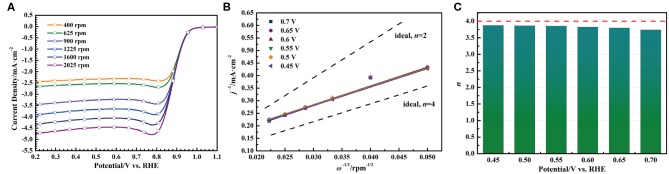
**(A)** LSV curves of the S-Co-N/C-I with different rotating speeds, **(B)** the corresponding K-L plots, and **(C)** the corresponding electron transfer number.

In order to investigate the kinetics of oxygen reduction reaction catalyzed by the S-Co-N/C-I, the Koutecky-Levich (K-L) method was used to analyze the oxygen reduction reaction process. As shown in Figure 6A, a set of LSV curves were first obtained by scanning the catalyst coated working electrode from 1.1 V to 0.2 V (vs. RHE) with a rate of 5 mV·s^−1^ at different rotating speeds of RDE (2,025, 1,600, 1,225, 900, 625, and 400 rpm). With the decrease of the RDE rotating speed, the diffusion current density in the LSV curve decreases gradually, which is caused by the increased transmission distance between the O_2_ saturated alkaline solution and the active site of the S-Co-N/C-I. And then, the current densities of each LSV curve at the potentials of 0.45, 0.50, 0.55, 0.60, 0.65, and 0.70 V (vs. RHE) were measured to obtain the K-L plots by Equation 3, as shown in Figure 6B. At last, the electron transfer number (*n*) in Figure 6C during the oxygen reduction reaction catalyzed by the S-Co-N/C-I can be calculated by using Equation 4.

(3) $$\begin{array}{l} \frac{1}{j}=\frac{1}{j_{\text{k}}}+\frac{1}{j_{\text{d}}}=\frac{1}{j_{\text{k}}}+\frac{1}{B\omega^{1/2}} \end{array}$$

(4) $$\begin{array}{l} B=0.2nFC_{{\text{O}}_{2}}{D_{{\text{O}}_{2}}}^{2/3}\mu^{-1/6} \end{array}$$

where *j*_d_ represents the diffusion current density, ω represents the disk rotating speed, *n* represents the electron transfer number, *F* represents the Faraday constant of 96,485 C·mol^−1^, *C*_O2_ represents O_2_ concentration of 1.21 × 10^−6^ mol·cm^−3^, *D*_O2_ represents the O_2_ diffusion coefficient of 1.86 × 10^−5^ cm^2^·s^−1^, and *v* represents the kinematic viscosity of 0.01 cm^2^·s^−1^.

In the potential range of 0.45~0.70 V (vs. RHE), the electron transfer number of the oxygen reduction reaction is in a range of 3.74~3.87, indicating that the kinetics process of the oxygen reduction reaction catalyzed by the S-Co-N/C-I is a four-electron pathway through the following four steps:

(5) $$\begin{array}{l} {\text{O}}_{\text{2}}\text{+}{\text{H}}_{\text{2}}\text{O+}{\text{e}}^{-}\text{+*}\to \text{OOH*+O}{\text{H}}^{-} \end{array}$$

(6) $$\begin{array}{l} \text{OOH*+}{\text{e}}^{-}\to \text{O*+O}{\text{H}}^{-} \end{array}$$

(7) $$\begin{array}{l} \text{O*+}{\text{H}}_{\text{2}}\text{O+}{\text{e}}^{-}\to \text{OH*+O}{\text{H}}^{-} \end{array}$$

(8) $$\begin{array}{l} \text{OH*+}{\text{e}}^{-}\to \text{*+O}{\text{H}}^{-} \end{array}$$

where ^*^ represents the active sites of the S-Co-N/C-I.

## Conclusions

In this work, the effect of the sulfurization process on the microstructure and oxygen reduction catalytic activity of the S, Co, N doped carbon material with ZIF-8, and ZIF-67 as precursors are investigated. The obtained material of the S-Co-N/C prepared by a sulfurization-pyrolysis process shows a polyhedral composite porous structure. Most of the Zn elements in the precursor of ZIF-7 can be vapored out to generate the micropores for the final product. The Zn elements remaining in the material after the sulfurization would cooperate with Co elements to generate carbon nanotubes on the surface of the carbon material. The CoS shells produced by the prior sulfurization can protect the inner Co atoms from agglomeration during the after pyrolysis process, which promotes a uniform dispersion of Co elements. In addition, the pyridinic N can be selectively created through the sulfurization-pyrolysis process of ZIF-8 and ZIF-67. Owing to the accelerated oxygen adsorption and electron transfer on the surface of the material and on increased and homogeneously dispersed active sites, S-Co-N/C material prepared by the sulfurization-pyrolysis process performs an enhanced ORR catalytic activity.

## Data Availability Statement

The datasets generated for this study are available on request to the corresponding author.

## Author Contributions

B-YS and SY contributed the same to this article and co-wrote the manuscript. B-YS conducted the synthesis of the materials. SY carried out the characterization and the electrochemical measurements.

## Conflict of Interest

The authors declare that the research was conducted in the absence of any commercial or financial relationships that could be construed as a potential conflict of interest.
