# Supplementary material for: Optimization of Sulfurization Process of Cobalt Sulfide and Nitrogen Doped Carbon Material for Boosting the Oxygen Reduction Reaction Catalytic Activity in Alkaline Medium
Source: Front Chem. 2020 Apr 28;8:314. doi: 10.3389/fchem.2020.00314 (PMC7199712; doi:10.3389/fchem.2020.00314)
Supplement: Supplementary file 1 [file Table_1.DOCX]

Supplementary Material

**Table S1.** Metal elements content of the obtained S-Co-N/C materials

| S-Co-N/C | Zn/wt.% | Uncertianty of Zn/wt% | Co/wt.% | Uncertianty of Co/wt% |
| --- | --- | --- | --- | --- |
| S-Co-N/C-I | 2.87 | 0.13 | 12.87 | 0.60 |
| S-Co-N/C-II | 0.20 | \ | 12.37 | 0.67 |
| *The Zn and Co elements of S-Co-N/C-II are cited from (Song et al., 2020) | | | | |

**Table S2.** Binding energy of Zn, C, S, N, and Co at the deconvoluted peak

| Element | Deconvoluted Peak | Binding Energy/eV | |
| --- | --- | --- | --- |
|  |  | S-Co-N-C-I | S-Co-N-C-II |
| Zn | Zn 2p_3/2_ | 1021.5 | \ |
|  | Zn 2p_1/2_ | 1044.6 | \ |
| C | C-C | 284.8 | 284.8 |
|  | C-N | 285.4 | 286.0 |
|  | C-O | \ | 288.6 |
| N | Pyridinic N | 398.3 | 398.3 |
|  | Co-N | 400.1 | 399.1 |
|  | Pyrrolic N | 401.4 | 400.4 |
|  | Graphitic N | 403.9 | 401.4 |
| S | S 2p_3/2_ | 161.9 | 162.3 |
|  | S 2p_1/2_ | 162.7 | 162.9 |
| Co | Co^0^ | 778.7 | 778.7 |
|  | Co^2+^/Co^3+^ | 781 | 781.7 |

**Table S3.** The Specific surface area of the obtained materials of S-Co-N/C

| Sample | Preparation process | Specific surface area/m^2^·g^-1^ |
| --- | --- | --- |
| S-Co-N/C-I | Sulfurization-pyrolysis | 369.1 |
| S-Co-N/C-II | Pyrolysis-sulfurization | 377.8 |

**Table S4.** The ORR catalytic activity of the obtained S-Co-N/C materials

| Samples | Onset potential (vs. RHE)/V | Half-wave potential  (vs. RHE)/V | Tafel slope/mV·dec^-1^ |
| --- | --- | --- | --- |
| S-Co-N-C-I | 1.009 | 0.879 | 59 |
| S-Co-N-C-II | 1.009 | 0.868 | 68 |

**References:**

Song, B.Y., Li, M.J., Yang, Y.W., He, Y.L. (2020). Achievement of a novel porous non-noble-metal catalyst with excellent oxygen reduction reaction activity: Promoting the commercialization of alkaline fuel cells. *J. Clean Prod.* 249, 119314. doi: 10.1016/j.jclepro.2019.119314
